# Quad Tendon Autograft for Posterior Cruciate Ligament Reconstruction Using Transseptal and Posteromedial Portals

**DOI:** 10.1016/j.eats.2023.02.003

**Published:** 2023-04-24

**Authors:** Victor Germon, Sylvain Guy, Alice Abs, Alexandre Ferreira, Christophe Jacquet, Jean-Noël Argenson, Matthieu Ollivier, Corentin Pangaud

**Affiliations:** a Public Teaching Hospital of Marseille, Marseille, France; b Aix-Marseille University, Marseille, France; c Institute of movement and locomotion, Marseille, France; d Public Teaching Hospital of Caen, Caen, France

## Abstract

We describe a surgical technique for reconstruction of the posterior cruciate ligament with quad tendon autograft using transseptal portal. We place the guide for the tibial socket through the posteromedial portal instead of transnotch, which is the most common practice. The use of the transseptal portal allows good visualization during the drilling of the tibial socket to protect the neurovascular bundle while avoiding the use of fluoroscopy. The advantage of using the posteromedial approach is the easy placement of the drill guide and to option to pull the graft once through the posteromedial portal and a second time through the notch, which helps passing the “killer turn.” The quad tendon is harvested with a bone block that is placed in the tibial socket and fixed with screws in the tibial and femoral side.

Posterior cruciate ligament (PCL) injuries are uncommon and usually occur in the context of combined ligamentous knee injury. Isolated PCL tears account for approximately 3% of acute knee injuries.^1^ Surgical reconstruction of the PCL is technically demanding. Potential challenges include visualization of the tibial footprint and drilling of the tibial tunnel without damaging posterior neurovascular structures, as well as graft selection, deployment, tensioning, and fixation

The PCL is an anisometric double-bundle structure. However, a meta-analysis showed comparable clinical and functional outcomes regarding single- versus double-bundle PCL reconstruction.^2^ We present a single-bundle PCL reconstruction using a transseptal portal arthroscopy. Our preferred graft is the quadriceps tendon autograft,^3^ with bone block. The allograft can be used for the multiligament reconstruction.

## Surgical Technique (With Video Illustration)

Informed consent from the patient was required before performing the surgery.

### Patient Position

**Fig 1:**
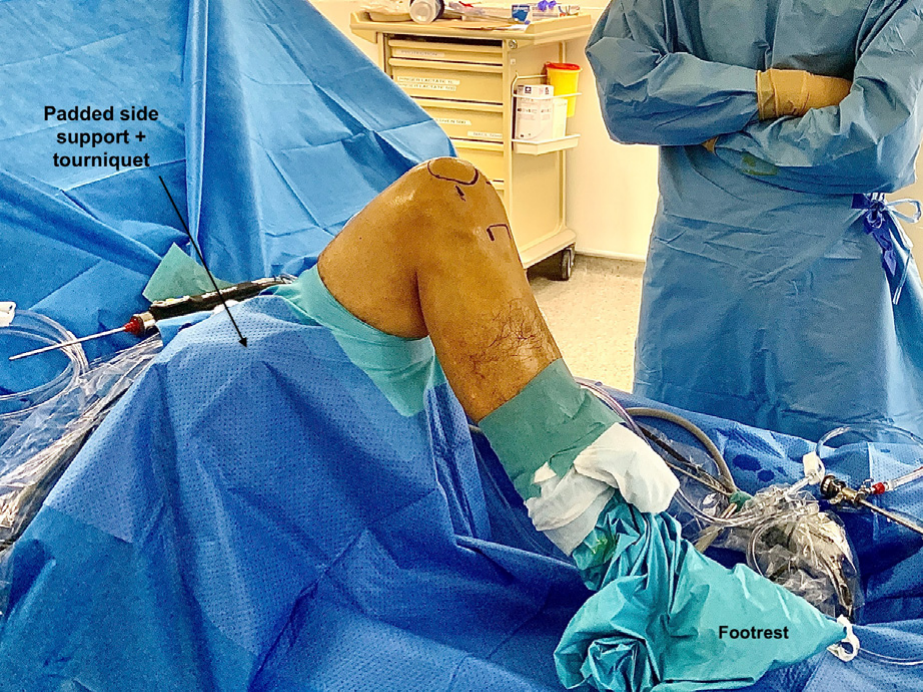
Patient positioning: The patient is positioned supine with the operative knee flexed at 90° (Left knee). A padded side support, footrest, and a thigh tourniquet are used.

The patient is positioned supine on a standard table with the operative knee flexed at 90°. A padded side support and footrest are used, and a thigh tourniquet is inflated throughout the surgery. A double-flow arthroscopy system may be used to avoid the use of a tourniquet. The fluoroscopy system is present in the room if necessary (Fig 1).

### Graft Harvest and Preparation

**Fig 2:**
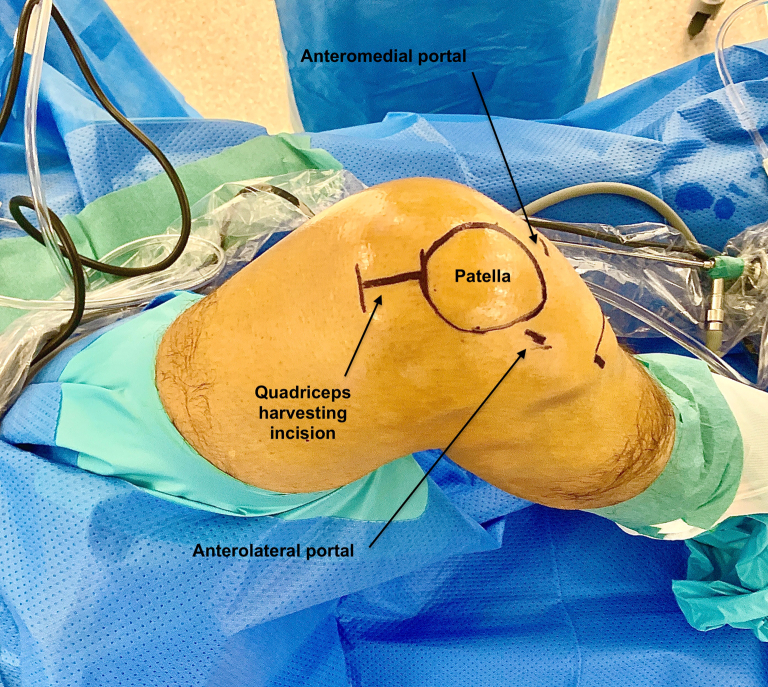
Incision for quad tendon harvesting: 3 cm from the top of the patella (Left knee).

The quadriceps tendon is harvested through a 4-cm incision at the proximal pole of the patella (Fig 2).^4^ A bone plug measuring 2 cm long and 1 cm width is withdrawn. A graft of 1 cm width is removed with a double scalpel, and the length of the tendon graft is approximately 10 cm. The tendinous part of the graft is whipstitched, and the bony part of the graft is drilled to pass a nonresorbable suture. The average diameter of the graft should be 10 mm.

### Arthroscopy Portals

A high anterolateral portal is first created adjacent to the patellar tendon. A low anteromedial portal, also adjacent to the patellar tendon, is then established under direct vision, in line with the ACL. A standard posteromedial (PM) portal is created under direct vision by use of the arthroscope through the anterolateral portal. From the PM portal, shaver is introduced to the septum, tangential of the posterior part of femoral condyle and parallel to the joint line.

**Fig 3:**
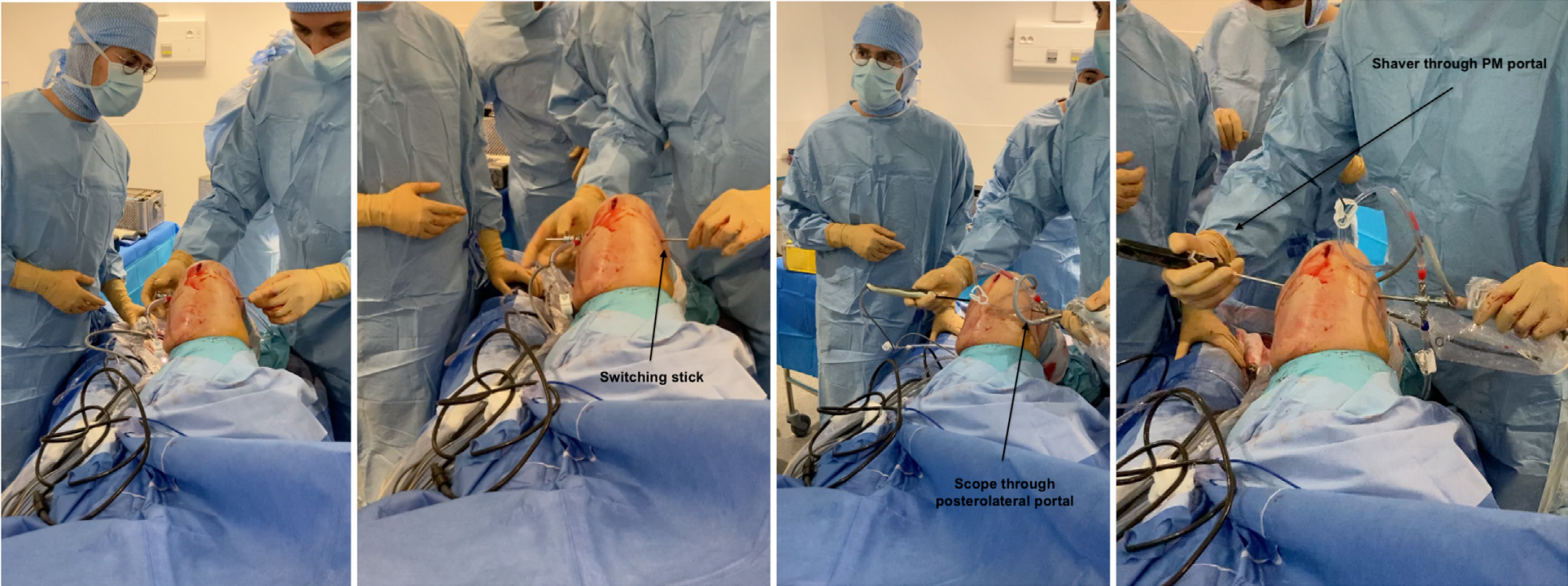
The posteromedial portal is created first; the septum is shaved under control of the view and the scope is then passed through the septum to create the posterolateral portal (Right knee, supine position, knee flexed at 90°).

The arthroscope is kept through the anterolateral portal while the shaver creates the portal through the septum under control of the view.^5^ A switching stick is passed through the posterolateral (PL) portal, while staying in touch of posterior part of condyle (Fig 3). A 1-cm incision is done on the switching stick. The sleeve is then put on the switching stick using the posterolateral portal to put the scope in a lateral view at the back of the knee.

### Femoral Socket Preparation

**Fig 4:**
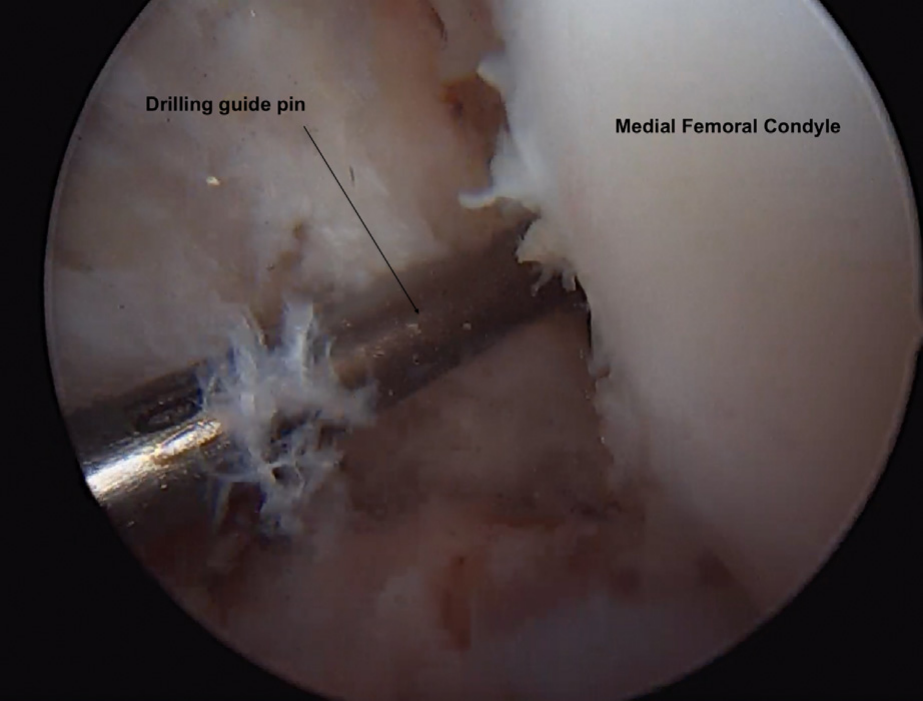
Femoral socket arthroscopic view: Intercondylar notch, medial condyle, 1 mm proximal to the cartilage, 10-o’clock location for the left knee and 2-o’clock location for the right knee (View from the anterolateral portal).

From the anteromedial portal, we drill the in-out femoral socket with a femoral offset drill guide (Versitomic; Stryker, Kalamazoo, MI). The guide is chosen according to the diameter of the graft: half of the diameter + 1 mm. The landmarks of the femoral socket are as follows: the intercondylar notch, medial condyle, 1 mm proximal to the cartilage, 10-o’clock location for the left knee and 2-o’clock location for the right knee.^6^ A first drill of 5 mm in diameter is performed, and a second drill is done with the diameter corresponding to the size of the graft. The depth of the socket is 25 mm (Fig 4).

### Tibial Socket Preparation

**Fig 5:**
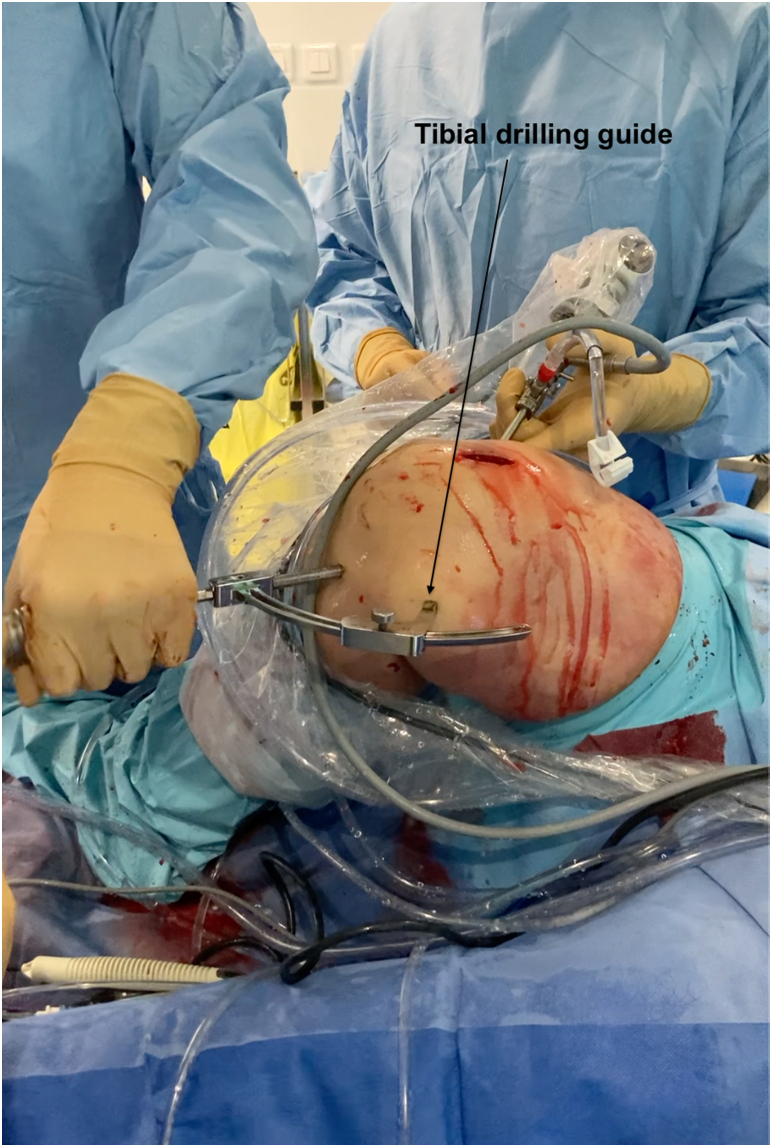
Using the posteromedial portal to place the tibial posterior cruciate ligament drilling guide allows easier positioning of the tibial socket (Right knee, supine position, knee flexed at 90°).

**Fig 6:**
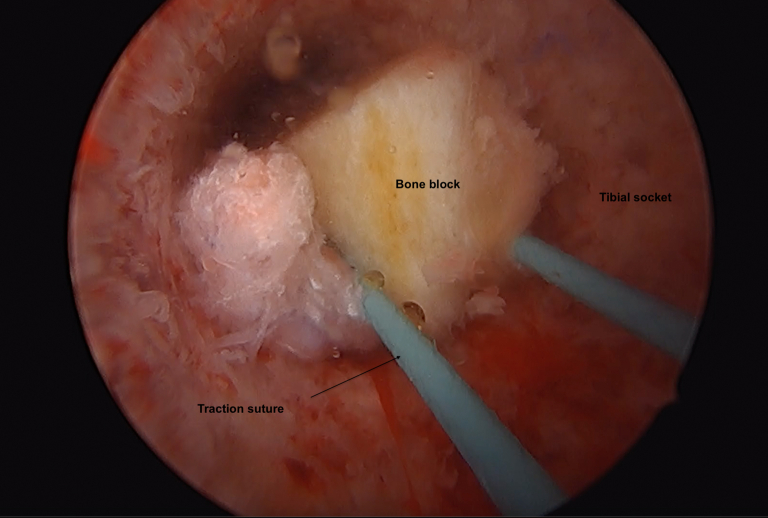
View of the bone block in the tibial tunnel (Scope placed in the tibial socket).

From the PL portal, the footprint of native PCL is prepared using electrocautery (SERFAS; Stryker). The footprint is located at the posterior part of the tibial plateau, 1 cm distal to the joint line posteriorly to the posterior horn of the lateral meniscus. The scar tissue of PCL is shaved, and the remnant of PCL is kept.^7^ The lateral limit of the tibial socket is the popliteal muscle. We use a classic tibial drill guide inserted through the PM portal (PCL Toolbox; Arthrex, Naples, FL; Fig 5). The angulation of the drill guide can be adjusted by the surgeon. The outside entrance of the socket stands 6 cm below the joint line and is placed medial to the tibial crest, proximal to the hamstring’s insertion. We drill an out-in tibial socket. The arthroscope through the PL portal allows the visualization of the extremity of the reamer; a curette may be placed through the PM portal to prevent overpenetration and potential damage of popliteal artery (Fig 6).

### Graft Deployment, Fixation, and Tensioning

**Fig 7:**
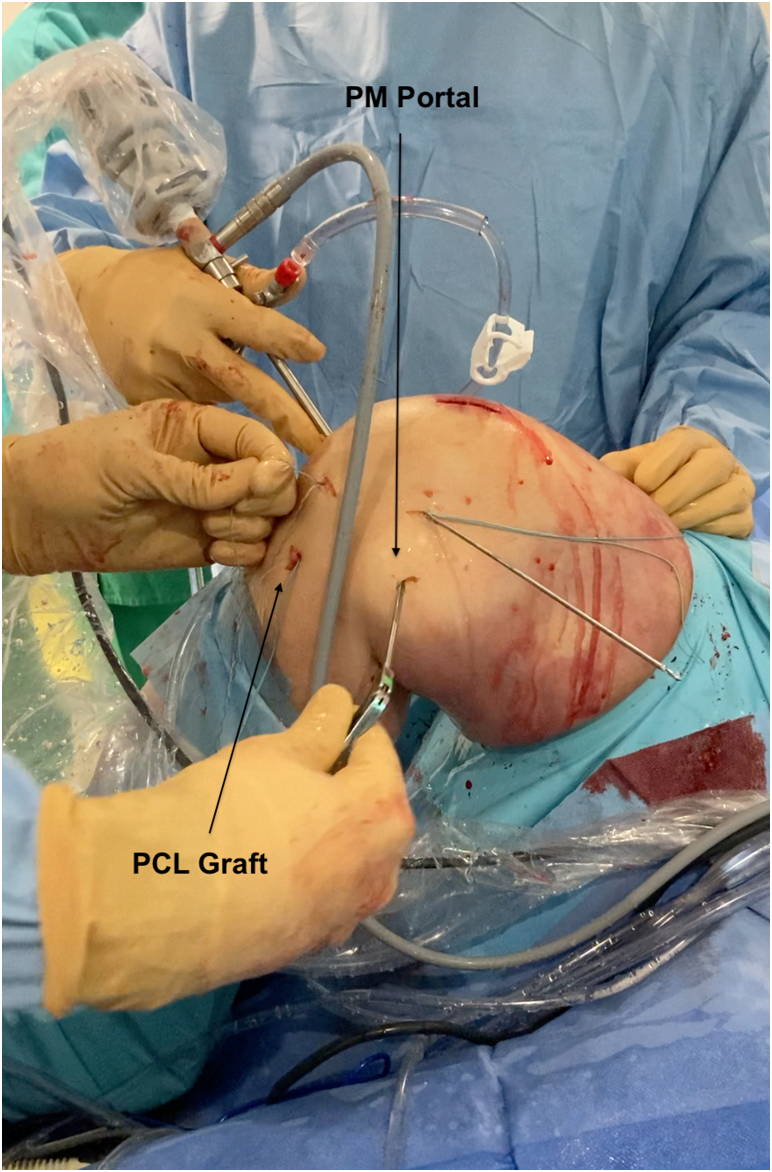
The posteromedial (PM) portal is used to pull the traction suture to avoid the killer turn while passing the graft from the tibial socket to inside the joint (Right knee, supine position, knee flexed at 90°). (PCL, posterior cruciate ligament.)

The graft is passed from the tibial tunnel and is fixed first in the femoral socket with the knee flexed to 90°. The PM portal is used to pull the traction sutures to avoid the killer turn (Fig 7). The fixation in the femoral socket is done with a bioabsorbable screw (FastThread; Arthrex) passed from inside the knee because the femoral socket is blind and drilled with an in-out guide. The diameter of the femoral screw is the same as the diameter of the socket to fix the soft part of the graft. The graft is fixed in the tibial socket with the knee flexed to 90° and anteroposterior constraint to correct the posterior drawer. The tibial fixation is done with a bioabsorbable screw (FastThread; Arthrex). The bone block is placed in the tibial tunnel and the diameter of the screw should be 1 mm below the diameter of the socket (Video 1).

### Postoperative Rehabilitation

Patient are kept non–weight-bearing for the first 30 days; knee flexion is allowed from 0 to 90°.

In this 30-day period, the patient wears a mobile PCL brace.

## Discussion

Various technique have been published concerning PCL reconstruction for example double-bundle reconstruction as in the study of Ponzo et al.^8^ and Yasen et al.,^9^ even though no functional differences have been published comparing single-bundle and double-bundle grafts.^10^^,^^11^ All-inside techniques have been published, with retro-drilling techniques allowing bone preservation in femoral and tibial sockets.^12^, ^13^, ^14^ Studies concerning the choice of the graft have shown no difference between hamstrings, quad tendon, or bone–patellar tendon–bone graft^15^ in the case of autograft. Allograft appears to be a reliable option, as Belk et al.^16^ reported no clinical differences when comparing autograft and allograft in their meta-analysis. Finally, more unusual techniques have been published, such as PCL repair^17^^,^^18^ or PCL repair with suture tape augmentation.^19^

The transseptal approach was first described by Ahn et al.^20^ in 2003 for PCL reconstruction. It recently was published as a technique using the transseptal portal with remnant preservation by Lee et al. in 2017^21^ for PCL reconstruction and for PCL cyst excision by Abreu et al.^22^ The 2 main innovations that our technique provides are the use of the quad tendon for PCL reconstruction and the use of posteromedial portal to place the PCL guide easily to drill the tibial socket.

This technique has several advantages: no fluoroscopy is needed to place the guide drilling pin, and the placement of the tibial socket is optimal because it is performed under view control unlike the technique using fluoroscopy. Using the posteromedial approach to place the PCL guide allows for easier positioning of the tibial footprint and good protection of the neurovascular bundle^23^ using a protective tool through this portal while drilling the socket. This portal is also used to avoid the killer turn while pulling the graft out of the tibial socket with a grasper.^24^ The risk is that performing a transseptal approach is highly difficult and should be performed by a trained sports medicine surgeon. The main dangers are due to the proximity of the neurovascular bundle, which can be damage by electrocautery. The learning curve should be started by performing the approach on cadavers according to the risks for the patients. The last disadvantage is the creation of an additional portal, which could increase the morbidity: the common fibular nerve could be damaged if the lateral portal is not placed in the right spot (Table 1).

**Table 1:** Advantages and Disadvantages

Advantages
No need for fluoroscopy
Optimal placement of the tibial socket
Easier positioning of the drilling guide
Avoids the killer turn
Protection of the neurovascular bundle
Disadvantages
Risky dissection near the neurovascular bundle
Technically demanding: learning curve
Creation of additional portals: posterolateral and posteromedial
Risk of nerve injury for the lateral portal and posteromedial portal

Finally, here are some tips and tricks that could be useful to the surgeon while performing this technique. Using arthroscopic electrocautery allows easier preparation of the tibial footprint. A fluoroscopy system should be in the operating room, ready to use in case of problems with the transseptal approach. We advise to perform a blind socket at the femur of 25 mm with the out-in technique to keep enough length of the graft before fixing it. Using the posteromedial portal to pull the graft helps to avoid the killer turn. Fixation should be performed first at the femur after confirming that sufficient graft is still in the tibial socket. We recommend placing the bone block on the tibial side and, if needed, performing double fixation with a double row anchor (Table 2).

**Table 2:** Pearls and Pitfalls

Pearls
Use arthroscopic electrocautery to prepare the tibial footprint
Have the fluoroscopy ready in case of problems
Perform a blind socket at the femur
Pitfalls
If the graft is too short, it might come out of the tibia while fixing at the femur
Do not pull the graft through the notch at first but use the posteromedial portal to avoid the killer turn

In conclusion, we believe that using the transseptal portal for PCL reconstruction is a reliable option associated with the quad tendon autograft. We describe the use of the posteromedial approach to place the drilling guide, using this approach to protect the neurovascular bundle and to pull the graft out of the socket. In our technique, a transnotch drill guide was used; we could also imagine designing a special drilling guide for the posteromedial portal that would make the technique even easier.

## Supplementary Data

ICMJE author disclosure forms

Video 1 Quad tendon autograft for posterior cruciate ligament reconstruction using transseptal and posteromedial portals is shown (Right knee).
